# Growth promotion and mycorrhizal colonization of Argan (*Argania spinosa* (L.) Skeels) inoculated with the edible desert truffle *Tirmania nivea* (Desf.) Trappe

**DOI:** 10.7717/peerj.13769

**Published:** 2022-08-17

**Authors:** Ahlem Khrizi, Fatima El-Houaria Zitouni-Haouar, Zohra Fortas

**Affiliations:** Laboratoire de Biologie des Microorganismes et de Biotechnologie, Département de Biotechnologie, Faculté des Sciences de la Nature et de la Vie, Université Oran 1 Ahmed Ben Bella, Oran, Algérie

**Keywords:** Desert truffles, *Tirmania*, *Argania spinosa*, Endomycorrhiza, Host plant growth

## Abstract

This study presents the first evidence of the mycorrhizal compatibility between the edible desert truffle *Tirmania nivea* and the valuable fruit tree *Argania spinosa*. Seed germination trials demonstrated that soaking pre-treatment of argan seeds in hydrogen peroxide (9%) for five days combined with the application of a fungicide treatment on an inert sowing material maximized the seed germination of this tree species. The mycorrhizal synthesis was conducted under greenhouse conditions by inoculating, *in vivo*, the host plant seedlings with spores of *T. nivea*. The growth and mycorrhizal status of *A. spinosa* was assessed 15.5 months after inoculation. The desert truffle mycorrhization significantly promoted all the investigated morphological parameters of growth and improved the physiological performances of the host plant through enhancing plant water status and chlorophyll concentration. The mycorrhizal symbiosis led to the formation of typical desert truffle endomycorrhizae with intracellular coils. The resistance of *A. spinosa* to the harsh environmental conditions of desert habitats makes it a potential candidate for cultivation of desert truffles.

## Introduction

Desert truffles are a vast family of hypogeous ascomycetes fungi delimited to taxa members of the genera *Terfezia, Tirmania, Picoa, Carbomyces, Elderia, Eremiomyces, Kalaharituber, Mattirolomyces, Mycoclelandia, Stouffera* and *Ulurua* (Kovács & Trappe, 2014). They are endemic to semi-arid and arid areas, mostly the countries around the Mediterranean basin, North-Africa and the Middle East. Desert truffles constitute an important part of the mycological flora of Algeria where they are represented by three genera *Terfezia, Tirmania* and *Picoa* (Fortas & Chevalier, 1992; Zitouni-Haouar, Fortas & Chevalier, 2014; Zitouni-Haouar et al., 2015; Zitouni-Haouar et al., 2018). These seasonal mushrooms cannot be regarded as a predictable crop since their fructification is strongly conditioned by a certain threshold of precipitation. Their ascomata (fruiting bodies) usually appear in December and they can still be harvested until the end of June.

Species of the genus *Tirmania* belong to a commercially important group of edible mushrooms with considerable socio-economic interest. Due to their delicate flavor, musky smell, and soft white tissues, these mushroom species are considered as the most highly prized desert truffles in the Middle East where they are better known as white truffles or *Zubaidi* (Al-Rahmah, 2001; Mandeel & Al-Laith, 2007). In good desert truffles seasons, *Tirmania* crops are marketed at prices comparable to those of meat, and served, indeed, instead of meat. However, in poor harvesting seasons, they become a very costly delicacy (Alsheikh & Trappe, 1983). Furthermore, *Tirmania* species have, undoubtedly, always been an important staple of the diet of several populations not only for their gastronomic value but also for their nutritional interest. In fact, they proved to be a rich source of proteins, amino acids, carbohydrates, fatty acids and minerals (Sawaya et al., 1985; Bokhary, 1987; Bokhary, Suleiman & Basalah, 1989; Hussain & Al-Ruqaie, 1999; Al-Ruqaie, 2009; Hamza, Jdir & Zouari, 2016; Bouatia et al., 2018). Nutritionally, digestible and non-digestible carbohydrates make *Tirmania nivea* a very healthy food providing both energy and fiber which are essential for the function of the intestinal track (Al-Laith, 2014). *Tirmania* also has a prominent place in the ethnomedicine of the Arabian countries as it is frequently used by Bedouins for the treatment of some ophthalmic diseases (Alsheikh & Trappe, 1983). These traditional medicine claims were then validated by several works which highlighted the therapeutic potential of *Tirmania* species through their interesting antimicrobial, antiviral, anticancer, antioxidant and antiradical activities (Chellal & Lukasova, 1995; Al-Laith, 2010; Gouzi et al., 2011; Gouzi, Leboukh & Bouchouka, 2013; Neggaz & Fortas, 2013; Stojkovic et al., 2013; Hamza, Jdir & Zouari, 2016; Gargano et al., 2017; Schillaci et al., 2017; Owaid, Muslim & Hamad, 2018; Elsayed et al., 2019a; Elsayed et al., 2019b).

*Tirmania* spp. are obligatory symbiotic mycorrhizal fungi. Their life cycle is only achieved through colonization of a suitable host plant, which is mainly an annual or perennial Cistaceae species belonging to the genus *Helianthemum*. Some affinities for a specific pH preferred by the host plant were also observed for this group of desert truffles. This is the case of the alkaline preference of *T. nivea* in accordance with its host plant *Helianthemum salicifolium*, while *Tirmania pinoyi* displays a preference for the acidophilous *Helianthemum* species, *H. guttatum * (Chevalier, 2014).

Several types of mycorrhizae have been described in previous desert truffles mycorrhizal associations investigated under natural or experimental conditions. Most of these involved *Terfezia* or *Picoa* species and emphasized the mycorrhizal plasticity of desert truffles to form ectomycorrhizae, endomycorrhizae and even ectendomycorrhizae on roots of different phytosymbionts (Awameh, Alsheikh & Al-Ghawas, 1979; Awameh, 1981; Alsheikh, 1984; Chevalier et al., 1984; Dexheimer et al., 1985; Leduc, Dexheimer & Chevalier, 1986; Roth-Bejerano, Livne & Kagan-Zur, 1990; Fortas & Chevalier, 1992; Kagan-Zur et al., 1999; Gutiérrez, Morte & Honrubia, 2003; Kovács, Vágvölgyi & Oberwinkler, 2003; Zaretsky et al., 2006; Slama et al., 2010; Navarro-Ródenas et al., 2012; Navarro-Ródenas et al., 2013; Zitouni-Haouar, Fortas & Chevalier, 2014; Dafri & Beddiar, 2018). However, mycorrhizae of *Tirmania* species have been the least studied so far and only one work was conducted on the mycorrhizal status of *T. nivea,* reporting the formation of endomycorrhizae on *Helianthemum ledifolium* and *H. salicifolium* roots inoculated with this *Tirmania* species (Awameh, Alsheikh & Al-Ghawas, 1979). In light of these many experiments, several factors such as the fungal species (Chevalier et al., 1984), phosphorus substrate fertility (Fortas & Chevalier, 1992; Navarro-Ródenas et al., 2012), culture conditions (Gutiérrez, Morte & Honrubia, 2003), common mycorrhizal type of the host plant (Kovács, Vágvölgyi & Oberwinkler, 2003), auxin–phosphate interaction (Zaretsky et al., 2006), irrigation water availability (Navarro-Ródenas et al., 2013) or the host plant (Zitouni-Haouar, Fortas & Chevalier, 2014) were suggested to help explain structural variability of desert truffles mycorrhizae.

The symbiotic relationship between desert truffles and their host plants was the key for the domestication of these wild edible mushrooms through the production and field transplantation of the desert truffles-*Helianthemum* mycorrhizal seedlings (Slama et al., 2010; Morte, Honrubia & Gutiérrez, 2008; Morte et al., 2009; Morte et al., 2012; Morte et al., 2017; Morte, Gutiérrez & Ródenas, 2020). Since the first *Terfezia* ascomata were produced from a plantation of *Helianthemum almeriense* mycorrhizal plants in Spain, an increasing demand for this crop has prompted research into new strategies to enhance the production of these fungi. This will be possible by the selection and propagation of suitable mycorrhizal seedlings adapted to the cultivation sites (Morte & Andrino, 2014).

The cultivation of *Terfezia* spp. and *Tirmania* spp. is also compatible with woody crops like almond, cherry tree and olive trees, among others, to optimize land use and irrigation systems. This allows a double crop in the same plot (one tree-borne the other—hypogeous truffles) (Honrubia, Andrino & Morte, 2014). Although desert truffles were mostly harvested under *Helianthemum* species, *Tirmania* ascomata were frequently collected in the desert plains of Algeria near to *Argania spinosa* trees (A. Khrizi, 2017, personal communication). This woody species is a fruit tree with an enormous economic importance related especially to oil production destined for nutritional, medicinal and cosmetic purposes (Zunzunegui et al., 2010). It is perfectly suited to arid environments and can grow on poor, shallow soils. Furthermore, owing to its deep rooting system, it is considered as having a strong effect against erosion and desertification (Nouaim et al., 2002) which are the main environmental problems encountered in desert truffles natural habitats. Thus, the main objective of the present study was to assess and characterize the mycorrhizal potential of *T. nivea* for establishing an effective mycorrhizal symbiosis with *A. spinosa* with the aim of involving this tree host species in desert truffle mycorrhizal plant production for cultivating these prized edible fungi. Moreover, this symbiotic interaction opens the possibility of a double-cropping (desert truffles—Argan fruits) in the same field. The mycorrhization with desert truffles is also a precious tool for improving the growth and survival of the nursery-grown Argan seedlings which is still a challenge in the reforestation programs of this valuable tree species within its area of distribution.

## Materials and Methods

### Origin of the fungal material

Mature desert truffles ascomata were collected in April 2011 from Bechar province (a Saharan area, Southwest of Algeria), near their natural host plant *Helianthemum lippii.* The ascocarps were first morphologically characterized and then sun-dried for 2 months. The desert truffles exsiccata were finally preserved at room temperature in paper bags.

### Morphological and molecular identification of the fungal material

Macromorphological characteristics including color, form and size of peridium and gleba were recorded from fresh ascomata. Micromorphological studies of asci and spores were conducted on rehydrated gleba sections cut from dried samples. Microscopic observations were performed in distilled water and Melzer’s reagent (Langeron, 1952). Ascospore and asci shape and dimensions were measured in distilled water mounts on at least 50 randomly selected mature spores using an Olympus CX22 microscope equipped with an ocular micrometer.

The most representative ascoma was chosen for the molecular characterization which was performed by sequencing the ITS region of the nuclear ribosomal DNA. The genomic DNA was extracted from 20 mg of exsiccata powder obtained after gleba grinding in liquid nitrogen using GF-1 Plant DNA Extraction Kit (Vivantis Technologies, USA) following the manufacturer’s instructions.

The polymerase chain reaction (PCR) amplification was carried out on the ITS1-5.8S-ITS2 region of the rDNA as described by Zitouni-Haouar et al. (2015), using the primers ITS1 and ITS4 (White et al., 1990). The amplification success was checked on 1% agarose gel stained with ethidium bromide. PCR products were sequenced with the same ITS primers at Eurofins Genomics (Ebersberg, Germany).

The sequence generated in this study was first compared with sequences deposited in public databases using the BLAST algorithm (Altschul et al., 1997), for its taxonomic affiliation and then deposited in GenBank (http://www.ncbi.nlm.nih.gov) under accession number MZ379289.

The morphological characterization and the BLAST analysis of the rDNA ITS sequences revealed the strong affiliation of the studied ascomata to the *Tirmania nivea* taxon.

### Plant material

The seeds used in the present study came from ripe fruits of similar size harvested in July 2015 from Argan trees “*A. spinosa*” in the region of Tindouf (Southwest of Algeria, 27°40′00″N, 8°09′00″W and 450 m altitude).

### Substrat used for the mycorrhizal synthesis

The soil used for the experiment was collected from Bechar (Southwest Algeria, 31°37′00″N, 2°13′00″W). It has been analyzed and found to present the following physico-chemical characteristics: pH = 7.12, organic matter = 0.563%, assimilable phosphorus = 83.47 ppm, N = 0.02%, AC = 0.5%, TC = 6.36%, EC = 0.201 ds/m. The soil texture analysis revealed its loamy-sand texture with mean composition of 80.6%, 15.16% and 4.24% for sand, silt and clay respectively. The collected soil was dried in the open air, sieved through a 2-mm mesh sieve to eliminate pebbles and large debris and then autoclaved for 1 h at 120 °C (Fortas, 1990).

### Mycorrhizal synthesis and assessment

### Seed germination tests of *Argania spinosa*

The Argan nuts containing the seeds were obtained by drying the fruits in the sun and then removing their pulp by hand (Nouaim et al., 2002; Benaouf, Miloudi & Belkhodja, 2014). The nuts were then stored in paper bags in the laboratory at ambient temperatures until they were sown. The argan seeds present hard shells that make the germination process complicated. In order to remedy this phenomenon, we have tested different combined seed treatments, inspired from several previous works, and based mostly on hydrogen peroxide and water soaking treatments which were applied directly on the nuts without removing their shells (Khelifi, Morsli & Khelifi-Slaoui, 1996; Berka & Harfouche, 2001; Nouaim et al., 2002; Miloudi & Belkhodja, 2009; Ikinci, 2014). The efficiency of the germination assays was also evaluated with the application of a fungicide SUMICO®, which was kindly provided by Dr. Gérard CHEVALIER. The different experiments conducted to enhance the Argan seed germination are indicated in Table 1. The germination was checked daily and evaluated on 100 seeds used for each experiment.

**Table 1:** Effect of different pre-sowing treatments on seed germination of Argan tree.

**Experiments**	**Prior chlorine disinfection treatment**	**Soaking solution**	**Soaking time**	**Soaking temperature**	**Sowing material**	**Sowing Temperature**	**Germination period**	**Cumulative seed germination %**	**Observations**
Experiment 1	Disinfection of seed coat with chlorine bleach for few seconds and washing	Sterile water	96 h	Room temperature (∼20 °C)	Sterile moistened cotton	30 °C	15 days	30%	Fungal contamination
Experiment 2	No prior disinfection	H2O2 (9.9%)	72 h	25 °C	Water agar medium	25 °C	10 days	7%	Seed rot
Experiment 3	No prior disinfection	H2O2 (9.9%)	96 h	25 °C	Water agar medium	30 °C	One week	18%	Seed rot
Experiment 4	No prior disinfection	H2O2 (9.9%)	One week	30 °C	Sterile moistened mold	Greenhouse temperature (∼20 °C–25 °C)	One month	48%	Seed rot
Experiment 5	No prior disinfection	H2O2 (9%)	120 h	30 °C	Sterile surgical compress humidified in permanence with SUMICO® fungicide treatment	30 °C	10 days	80%	Maximum germination rate without any rot or seed contamination

### Experimental mycorrhizal inoculation

The mycorrhizal synthesis was realized in 400 cm^3^ polyethylene pots filled with the sterilized soil and amended with well-germinated seeds (seed rootlet length ≈ one cm) sown directly on the surface of the substrate and covered with a thin soil layer equaling the seeds volume. Inoculation was realized with a mature aqueous spore suspension of *T. nivea* prepared by blending rehydrated carpophore pieces. For each pot (containing 1 seedling), an ascospore suspension containing 1 g of rehydrated fruiting body in 50 ml of sterilized distilled water and with a concentration of approximately 1–2.8 × 10^9^ spores was applied directly in contact with the seedling roots inside the pot according to the technique of Zitouni-Haouar, Fortas & Chevalier (2014). The production of mycorrhized plants was performed following the technique of Chevalier, Grente & Pollacsek (1973), Chevalier & Grente (1979) and Fortas & Chevalier (1992) applied for the mycorrhizal synthesis of *Tuber melanosporum* and desert truffles in genera *Terfezia* and *Tirmania* respectively. The pots containing uninoculated, control seedlings received 50 mL of distilled water instead of the ascospore suspension. Seedlings were then grown in non-conditioned greenhouse under natural lighting from March until their harvest in June of the next year (15 months and a half). The plants were watered during the first month of the experimentation with sterilized water and then were periodically irrigated when necessary with tap water.

### Mycorrhiza observation and characterization

After 15.5 months of culture, both the inoculated seedlings and non-inoculated controls were carefully removed from their pots and their root systems gently freed from soil particles by their immersion for a half-hour in individual water containers in order to minimize the loss or the physical disturbance of the mycorrhizal roots. The whole root systems were then examined using a stereomicroscope to identify the macro-morphological pattern of *T. nivea* mycorrhizae. The mycorrhizal type was subsequently determined under a light microscope on root pieces of 1-cm length randomly sampled from the root systems of inoculated seedlings. The root segments were prepared according to Phillips & Hayman (1970) protocol, modified slightly, with 2 h heating in 10% KOH clearing solution and 1 h staining at 90 °C in lactophenol-0.1% trypan blue. A randomly selected aliquot of fifty stained root fragments was then lightly rinsed with distilled water and mounted in lactoglycerol (v/v) on each of five glass microscope slides (10 per slide). The percentage of the mycorrhizal colonization was determined according to the frequency of infection (F (%)) which is expressed by: F % = 100 (N − N_0_)/N; where *N* is the total number of observed root fragments and N_0_ is the number of root fragments uninfected (Trouvelot, Kough & Gianinazzi-Pearson, 1986).

### Seedling growth assessment

At the end of the experimental period, ten seedlings randomly selected from each treatment were harvested for the evaluation of seedling growth. The effect of mycorrhization on plant growth was estimated by measuring the following morphological traits: plant height, leaf number, leaf length, shoot fresh weight, shoot dry weight and root fresh weight. The shoot dry weights were determined after oven drying the material at 60 °C in an air-forced oven for 72 h.

### Mycorrhizal growth response and mycorrhizal growth dependency

Mycorrhizal growth response and mycorrhizal growth dependency were estimated from the shoot dry weight of the inoculated and control plants. The mycorrhizal growth response (MGR) was calculated according to Hetrick, Wilson & Cox (1992) with the following equation: MGR % = [(dry weight of mycorrhizal plants − dry weight of non-mycorrhizal plants)/dry weight of non-mycorrhizal plants] × 100. The mycorrhizal growth dependency (MGD) was determined by expressing the difference between the average dry weight of mycorrhizal plants and the average dry weight of the non-mycorrhizal plants as a percentage of the dry weight of the mycorrhizal plants (Plenchette, Fortin & Furlan, 1983).

### Photosynthetic pigments concentrations

Chlorophyll a and b were estimated from the freshly harvested plants according to Navarro-Ródenas et al. (2013). The chlorophyllous pigments were extracted by grinding 0.05 g of leaf tissue in 5 ml of 80% aqueous acetone using a mortar and pestle at 4 °C. The resulting extracts were centrifuged at 10,000 *g* for 10 min and the absorbance of the solution was measured by UV–Visible spectrophotometry at 646 and 663 nm. The chlorophylls concentrations were then calculated using the equations of Wellburn (1994):

Chlorophyll a (C_a_) = 12.21 × A_663_ − 2.81 × A_646_

Chlorophyll b (C_b_) = 20.13 × A_646_ − 5.03 × A_663_.

The content of the total chlorophyll was expressed in milligram per gram (mg g^−1^) of fresh weight.

### Analysis of plant water status

Leaf relative water content (RWC) was estimated according to Barrs (1968). A composite sample of 10 leaf discs is taken and the fresh weight is recorded. The turgid weight is then determined after saturation of leaf tissue in distilled water for 24 h at 4 °C. The dry mass weight is subsequently evaluated after oven-drying the leaf samples to a constant weight at 80 °C for 24 h. Relative water content is calculated using the following formula: RWC (%) = (fresh weight − dry weight)/(turgid weight − dry weight) × 100.

Leaf hydration was determined according to Alessio et al. (2008) as H (%) = (FW − DW)/DW × 100; where FW is leaf fresh weight and DW is leaf dry weight. Dry weight was measured after drying leaves in an oven at 70 °C for 72 h (until constant weight was reached).

### Statistical analyses

The Morpho- and physiological data were analyzed using a Student’s *t*-test. Computations were carried out using the statistical software STATISTICA (version 10.0, StatSoft) for Windows. Differences among treatments were considered significant at *p* < 0.05.

## Results

### Evaluation of seed germination of *Argania spinosa*

**Figure 1:**
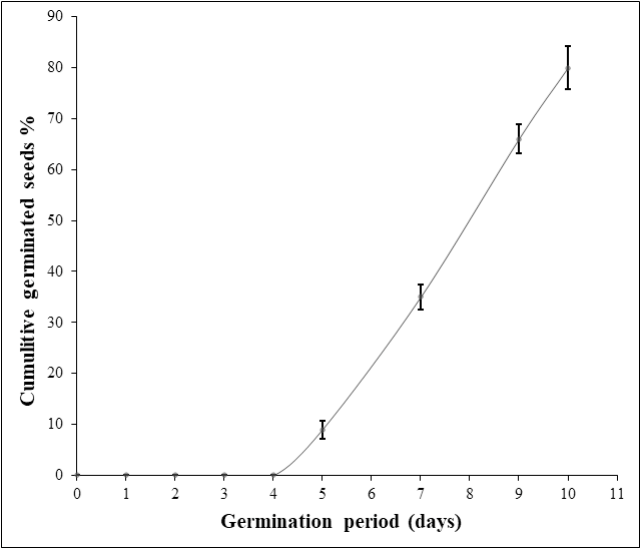
Daily Argan seed germination rate of the best experiment (experiment 5). The germination assay was performed on two seed batches (replicates) of 50 seeds each and the germination percentage values were used to calculate the standard deviation (SD).

Considering the first experiment, disinfection of seed coat with chlorine bleach followed by sterile water soaking and sowing on sterile moistened cotton led to a mediocre germination percentage (30%) with fungi-seed contamination. Applying hydrogen peroxide (9.9%) for seed surface-sterilization and as a soaking solution gave better results in terms of inhibition of fungal contamination. Nevertheless, this treatment resulted in seed rot except when a fungicide was applied. In the second and the third experiments, soaking seeds in hydrogen peroxide (9.9%) for 72h-96 h on water agar medium gave a very low germination rate (7%, 18%). Hydrogen peroxide soaking time was a significant source of variation for germination percentage and germination period. For experiment 4, the increasing of hydrogen peroxide soaking time to one week raised the germination percentage to 48%. However, the change in sowing conditions including mold as a sowing material and unstabilized ambient temperature extended the germination period to one month (Table 1). In the last experiment, the germination rate was significantly enhanced to the maximum (80%), ten days after seed soaking in hydrogen peroxide (9%) for 120 h at 30 °C combined with SUMICO® fungicide treatment on sterile moistened surgical compress. Thus, the use of a fungicide treatment suppressed seed deterioration and fungal contamination (Table 1; Fig. 1). Moreover, when both hydrogen peroxide (9%) and fungicide treatments were combined, seed dormancy was reduced considerably to five days (Fig. 1). It is important to notice that a germination rate of 100% was not achieved in any treatments, probably due to non-viability of some seeds.

### Morphology and frequency of the mycorrhizal colonization

**Figure 2:**
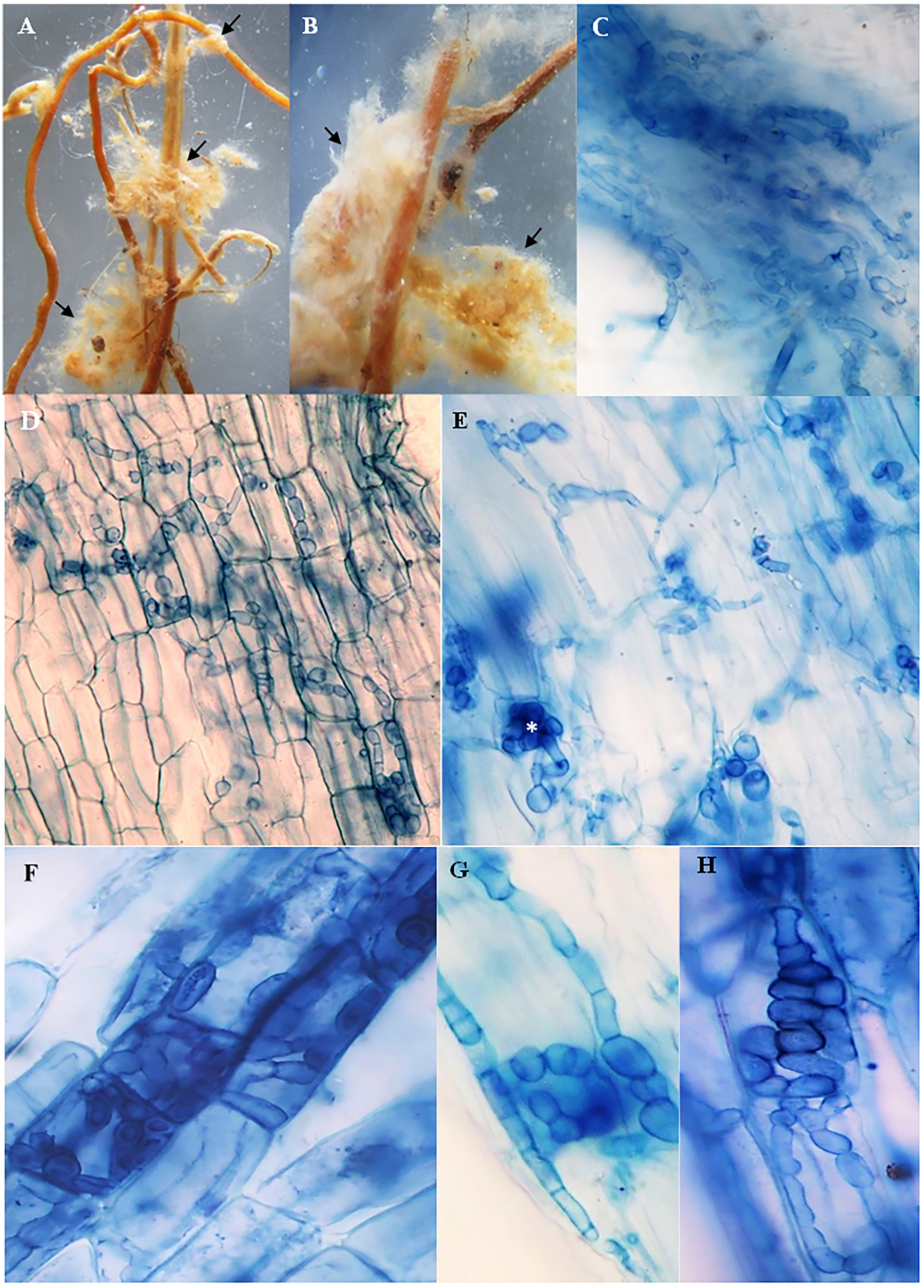
Mycorrhizal colonization of *Argania spinosa* roots inoculated with *Tirmania nivea*. (A, B) Macromorphological characters of mycorrhizal roots covered with compact yellowish mycelium masses, observed under stereomicroscope (arrows). (C) Inoculated roots invaded by dense *T. nivea* hyphae. (D–H) Intracellular colonization of cortical cells by *T. nivea* swelling hyphae forming coils (asterisks). All microscopic images ×640.

The roots of inoculated and non-inoculated seedlings of *A. spinosa* were examined fifteen months and a half after *in vivo* inoculation with *T. nivea*. Under stereomicroscope, the freshly collected inoculated roots appeared slightly swollen at the tips compared with uncolonized ones. Compact yellowish mycelium masses were also observed exclusively around the surface of the mycorrhizal roots (Figs. 2A and 2B). At the light microscope level, the cortical cells of the stained inoculated roots were colonized by hyphal coils and desert truffles endomycorrhizae. Indeed, *T. nivea* hyphae developed densely on the host root surface (Fig. 2C) and colonized intracellularly the inside of *A. spinosa* cortex cells by producing coiled hyphae (Figs. 2D and 2H). The *A. spinosa* inoculated roots were highly colonized; *T. nivea* mycorrhizae were detected in 74% of *A. spinosa* infected root samples (Table 2) whereas the control roots showed no evidence of mycorrhizal infection.

### Effects of mycorrhization on morpho-physiological growth parameters of the host plant

**Figure 3:**
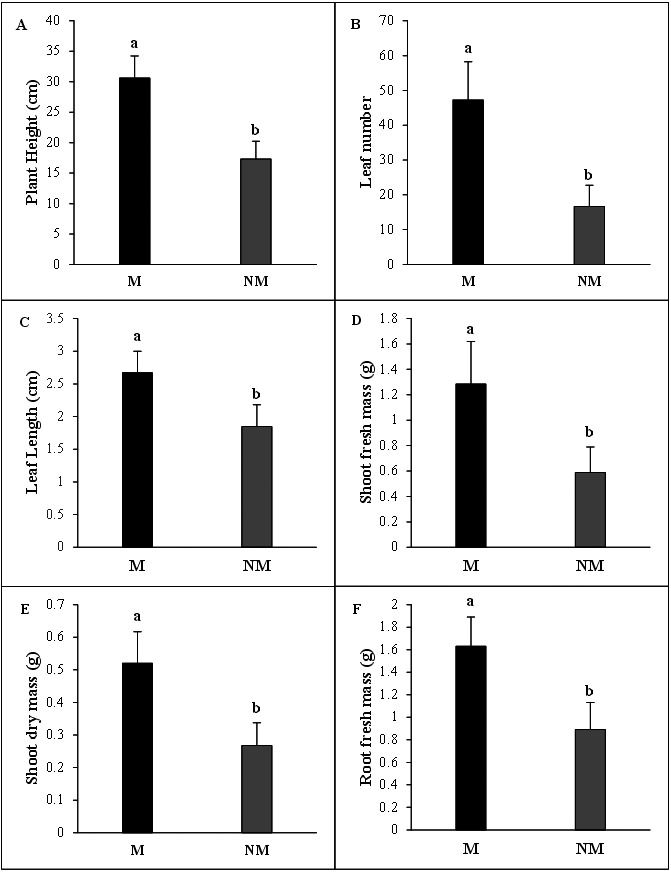
Effect of inoculation with *Tirmania nivea* on morphological growth parameters of mycorrhizal (M) and non-mycorrhizal plants (NM) of *Argania spinosa*. (A) Plant height, (B) leaf number, (C) leaf length, (D) shoot fresh weight (FW), (E) root fresh weight (FW), (F) shoot dry weight (DW).

The growth of *A. spinosa* mycorrhizal plants inoculated with *T. nivea* was significantly increased relative to that of uninoculated seedlings. The improvement in plant height was notably greater (*P* < 0.001) in inoculated plants than the controls. The number of leaves and mean leaf length were substantially increased in mycorrhizal plants by 2.83 and 1.45 folds, respectively, compared to the non-mycorrhizal ones. Moreover, the fresh green and the dry biomasses of the shoot and the density of the fresh root systems were sharply promoted by the twofold (*P* < 0.05) in inoculated plants in comparison with uninoculated control seedlings (Fig. 3). The high mycorrhizal growth response index (94.32%) highlighted the growth-stimulating effect of the mycorrhization by *T. nivea* on *A. spinosa* seedlings. In addition, the calculated value of the mycorrhizal growth dependency index (48.53%) reflected the positive response of *A. spinosa* to desert truffle mycorrhizal symbiosis (Table 2).

**Table 2:** Mycorrhizal colonization (MC), mycorrhizal growth response (MGR) and mycorrhizal growth dependency (MGD) of *A. spinosa* (AS) plants after 15.5 months of inoculation with *T. nivea* (TN).

**Combination**	MC (%)	MGR (%)	MGD (%)
AS/TN	74	94.32	48.53

**Figure 4:**
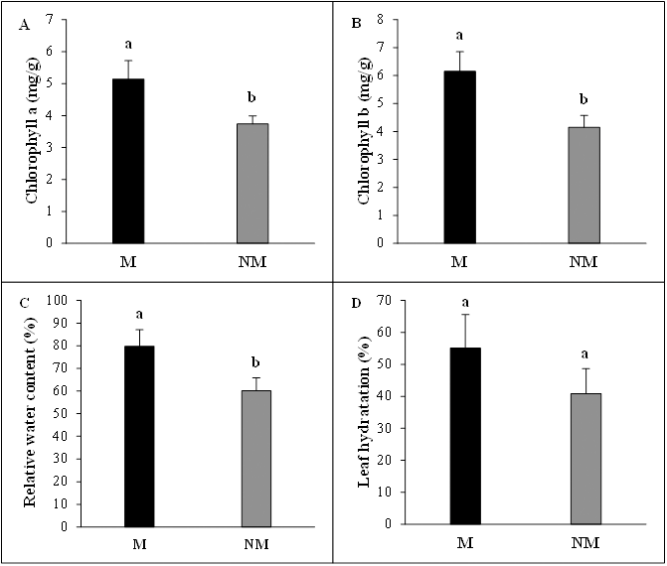
Effect of inoculation with *Tirmania nivea* on physiological growth parameters of mycorrhizal (M) and non-mycorrhizal plants (NM) of *Argania spinosa*. (A) Chlorophyll a content, (B) chlorophyll b content, (C) relative water content, (D) leaf hydration.

The mycorrhizal status had vital beneficial effects on photosynthetic pigments concentrations of *A. spinosa* seedlings. Chlorophyll a concentration per leaf fresh weight was significantly higher (*P* < 0.05) and increased by 37% in mycorrhizal plants compared to non-mycorrhizal plants. The chlorophyll b content in mycorrhizal plants presented a 48% increase above non-mycorrhizal ones, showing clearly once more the considerable contribution of mycorrhization to the improvement of photosynthesis activity in mycorrhizal seedlings. Moreover, the desert truffle mycorrhizal infection positively affected the tissue water status of *A. spinosa*. In fact, the leaves relative water content (RWC) values showed that the mycorrhizal plants contained ≈ 20% more water than controls and the leaf hydration amounts were higher by 14% in inoculated seedlings in comparison with non-inoculated ones (Fig. 4).

## Discussion

The present study highlighted the importance of the use of hydrogen peroxide (9%) instead of water as soaking pre-treatment at 30 °C combined with the application of an effective fungicide (SUMICO®) treatment on an inert sowing material for the success of Argan seed germination. The results of this work presented a simple, efficient and repeatable method ensuring a high argan seed germination rate and an enhanced seedling growth. It is important to point out that the success of seed germination and the establishment of vigorous seedlings in nurseries are crucial factors for the multiplication and propagation of *A. spinosa*, a multipurpose tree species of great economic, agronomic and ecological interests, but nevertheless, known for the complexity of its seed germination process.

The findings of this study clearly indicated that argan seed dormancy can be broken and germination is hastened using hydrogen peroxide (9%–9.9%) as a soaking pre-treatment at 30 °C. Furthermore, the seed germination increase seems to correlate well with the duration of soaking. Hydrogen peroxide plays a vital role in seed germination and plant growth development as well as the acquisition of stress tolerance (Li, Gong & Liu, 2012). Berka & Harfouche (2001) showed that hydrogen peroxide (3%) and warm water appear to be the best soaking pre-treatments to suppress the tegumentary inhibition of *A. spinosa* seeds. These authors demonstrated also that temperature appears to be a limiting factor in argan seed germination. Indeed, at high temperatures (25–28 °C), germinative rate and speed of germination are higher than at temperatures below 25 °C. The stimulating effect of hydrogen peroxide on seed germination has been also observed in several tree species such as *Pseudotsuga menziesii* (Ching, 1959), *Pinus roxburghii* (Ghildiyal, Sharma & Khanduri, 2007) or *Prunus scoparia* (Imani et al., 2011). On the other hand, the application of SUMICO® fungicide in this work appears to promote and speed up germination avoiding the deceleration and inhibition of germination engendered by the exposure of seeds to contamination. Moreover, the use of surgical compress seems to minimize fungal contamination and reinforce the fungicidal action. Our findings are in good agreement with the results of Bani-Aameur & Alouani (1999) who found that treatment with the fungicide thiram increased argan seed germination and its application is necessary to protect the nut, when the shell is scarified.

The mycorrhizal response of *A. spinosa* seedlings to infection by the desert truffle species *T. nivea* was assessed fifteen months and a half after *in vivo* inoculation. The mycorrhizal roots of *A. spinosa* were invaded by the irregular lobed septate hyphae of *T. nivea* which formed intracellular coils. These endomycorrhizal structures were discontinuously present throughout the whole colonized roots. The previous works investigating the mycorrhizal systems produced by desert truffles have reported their exceptional mycorrhizal plasticity. In fact, these fungi can form the three major types of mycorrhizae, *i.e.,* ectomycorrhizae, endomycorrhizae and ectendomycorrhizae either *in vivo* or *in vitro* culture conditions. However, this structural mycorrhizal variability was observed in response to biotic factors related to the mycosymbiont partner (Chevalier et al., 1984) or the phytosymbiont species involved in the mycorrhizal interaction (Zitouni-Haouar, Fortas & Chevalier, 2014) and its common mycorrhizal type (Kovács, Vágvölgyi & Oberwinkler, 2003). In addition, the desert truffles transition between ecto-, endo-, and ectendomycorrhizal types was also influenced by abiotic elements relevant to the level and the source of phosphorus in the culture substrate (Fortas & Chevalier, 1992; Navarro-Ródenas et al., 2012), the culture conditions of the mycorrhizal synthesis (Gutiérrez, Morte & Honrubia, 2003), the interdependency between exogenous phosphate and auxin levels (Zaretsky et al., 2006) or drought stress conditions (Navarro-Ródenas et al., 2013). This last factor is consistent with our suggestion that the bioclimatic origin of the host plant may determine the mycorrhizal type formed by desert truffle. Indeed, our experiment was conducted on *A. spinosa*, a host plant native to arid and desert bioclimatic niche well known by water scarcity conditions. Adaptation and surviving to water-stress episodes imply the adoption of a suitable mycorrhizal type allowing resistance to drought. Navarro-Ródenas et al. (2013) reported a morpho-physiological adaptation of *Terfezia claveryi-H. almeriense* symbiosis to drought conditions by forming endomycorrhizae. The results of these authors demonstrated that irrigation water scarcity induced a change in the mycorrhizal type formed by desert truffle, which was more intracellular under drought stress.

The high mycorrhizal colonization rate obtained in the present study provides strong evidence of the intimate interaction and the good mycorrhizal compatibility between *T. nivea* and *A. spinosa*. The common harvest of this desert truffle species close to *A. spinosa* trees in desert lands of Algeria also supports a probable mycorrhizal relationship between these two symbiotic partners in their natural biotope.

The *T. nivea* inoculation conferred significant developmental benefits to *A. spinosa* mycorrhizal seedlings compared to non-mycorrhizal ones. The results of the present work provide the first evidence of the positive effect of *T. nivea* mycorrhizae on the growth and development of a valuable fruit tree. Desert truffle mycorrhization successfully increased *A. spinosa* plant height, leaves number and length and green and dry biomasses. The data presented in this study are in perfect congruence with previous work performed on desert truffle mycorrhizal associations. The results of these investigations clearly demonstrated the positive correlation between root colonization by desert truffles mycorrhizae and enhanced growth attributes of the respective host plant most notably *Helianthemum* spp. In fact, Awameh (1981) reported an important increase in number of leaves and plant dimensions of the herbaceous *Helianthemum, H. salicifolium* and *H. ledifolium* plants inoculated by the desert truffle species *Terfezia boudieri*. In a similar work, *Helianthemum sessiliflorum-T.boudieri* mycorrhizal plants gained significantly more biomass, based on both fresh and dry weights, than did non mycorrhizal ones. The shoot to root ratio, (based on dry weights) was about twofold higher. Inoculation with *T. boudieri* enabled larger canopy development relative to root development, an outcome of the mycorrhizal support in the mycorrhized plants (Turgeman et al., 2011). Moreover, Zitouni-Haouar, Fortas & Chevalier (2014) demonstrated the significant beneficial effect exerted by the *in vivo* mycorrhization of *Terfezia leptoderma, T. boudieri* and *T. claveryi* on plant height, leaf number and length, and shoot dry matter values of several annual and perennial *Cistaceae* species from the genera *Helianthemum, Cistus* and *Fumana,* in addition to the forest tree species *Pinus halepensis.* However, some abiotic factors such as the phosphorus source or fungal secreted auxin seem to influence the growth response of the mycorrhizal host plants. Indeed, Navarro-Ródenas et al. (2012) investigated the effect of *T. claveryi* inoculation on the *in vitro* growth of *H. almeriense* with different phosphorus sources. A significant effect of the phosphorus source on the shoot growth was detected. Both phytate and inorganic phosphorus treatments reduced the shoot growth of the mycorrhizal plants compared to medium without any source of phosphorus. Turgeman et al. (2015) demonstrated moreover that *H. sessiliflorum* exposure to high levels of auxin secreted by *T. boudieri* at early stages of host development produced considerable slowing of seedling growth during the mycorrhizal establishment.

On the other hand, the high rate of the mycorrhizal growth response index obtained in the present study for *A. spinosa* mycorrhizal seedlings is a pure reflection of the nutritional benefits gained from mycorrhizae which are recognized as improved access to limiting soil resources, most notably immobile nutrients (*e.g.*, P, Cu, Zn, and ammonium) (Johnson, Graham & Smith, 1997). The abilities of mycorrhizal species and strains to promote plant growth opened new perspectives for the use of these fungi inoculations in nurseries and forestry (Martins, 2008). The mycorrhizal growth dependency (MGD) index value derived from this work (48.53%) is in good agreement with previous results obtained by Zitouni-Haouar, Fortas & Chevalier (2014) for a similar forest tree species *P. halepensis* (47.27%) mycorrhized by *T. leptoderma*. However, these authors found much higher values of MGD for other herbaceous and woody *Cistaceae* species. The mycorrhizal dependency or “responsiveness” was defined earlier by Gerdemann (1975) as the degree to which a plant species is dependent on the mycorrhizal condition to produce its maximum growth at a given level of soil fertility.

Inoculation with the desert truffle *T. nivea* and subsequent mycorrhizal formation contributed effectively to the improvement of the physiological performance of *A. spinosa.* Chlorophyll contents were considerably enhanced in the leaves of mycorrhizal plants compared to controls. This is particularly observed for chlorophyll b concentrations which were much higher in mycorrhizal plants than those of chlorophyll a. These results are in agreement with previous studies showing the significant enhancement in chlorophyll concentrations of desert truffles*-Helianthemum* spp. mycorrhizal seedlings than in controls (Turgeman et al., 2011; Navarro-Ródenas et al., 2013) with a specific increase in chlorophyll b content compared to chlorophyll a (2.4- and 1.52-fold, respectively) (Turgeman et al., 2011). The enhanced competence of mycorrhizal plants to persevere in the harsh environmental conditions of deserts is the outcome of special adaptations, including increased Chl b content, lower photosynthetic activation energy, and enhanced stomatal conductance, that alter and improve the physiological performances of the host plant (Turgeman et al., 2011).

Water status plays a major role in the growth and physiological processes of plants. Mycorrhizal and non-mycorrhizal plants frequently exhibit differences in water status (Augé, 2001). RWC and leaf hydration represent important criteria for the evaluation of plant water status. RWC is a useful indicator of the state of water balance of a plant essentially because it expresses the absolute amount of water, which the plant requires to reach artificial full saturation (González & González-Vilar, 2001). In this study, mycorrhizal plants exhibited better RWC values than their corresponding non-mycorrhizal plants. This finding showed that *T. nivea* fungal hyphae increased the water uptake of *A. spinosa* mycorrhizal roots which could further substantiate the potential of this host plant to grow and survive in the severe environmental conditions of its desert habitat. The improvement of water status *via* mycorrhizal symbiosis could play an indirect role in enhancing nutrient uptake, osmotic adjustment and the capacity for gas exchange (Zhu, Song & Xu, 2010). Our data are consistent with an earlier work performed by Turgeman et al. (2011) who reported higher rates of transpiration in *H. sessiliflorum* plants inoculated by *T. boudieri* than in control plants reflecting, thus, an increase in water uptake of mycorrhizal seedlings. Desert truffle mycorrhizae are also well known for increasing effective root hydraulic conductivity and survival rate of mycorrhizal plants under drought-stress conditions (Morte, Lovisolo & Schubert, 2000), providing the plant a greater capacity to tolerate limited soil water availability (Morte, Navarro-Ródenas & Nicolas, 2010).

## Conclusion

This work demonstrated the success of the *in vivo* mycorrhizal association between the highly prized edible mushroom *T. nivea* and the valuable host tree *A. spinosa.* The symbiotic association exerted a beneficial effect on the growth and physiological parameters of the host plant. The mycorrhizal compatibility of Argan tree with desert truffles and its strong adaptation to the hostile conditions of the desert truffles arid biotopes make it a potential phytosymbiont for the cultivation projects of these widely appreciated edible mushrooms while it can contribute simultaneously to afforestation and ecosystem restoration of its native degraded habitats.

##  Supplemental Information

10.7717/peerj.13769/supp-1 Supplemental Information 1 Tirmania nivea rDNA ITS FASTA sequence MZ379289

10.7717/peerj.13769/supp-2 Supplemental Information 2 Tirmania nivea ITS_Sequence MZ379289 This is an ab1 file (ABI sequencer data file) of the Tirmania nivea rDNA ITS sequence. This file can be viewed with the chromas software which is freely available on the link: http://technelysium.com.au/wp/chromas/

10.7717/peerj.13769/supp-3 Supplemental Information 3 Raw Data Daily germination rate

10.7717/peerj.13769/supp-4 Supplemental Information 4 Raw data Morphological growth parameters (height, leaf number, leaf length, shoot fresh weight “FW”, shoot dry weight “DW” and root fresh weight “FW”), and physiological growth parameters (chlorophyll a content, chlorophyll b content, relative water content, leaf hydration,) of *A. spinosa* (AS) plants after 15.5 months of inoculation with *T. nivea* (TN) (Raw Data)

10.7717/peerj.13769/supp-5 Supplemental Information 5 Student’s *t*-test analysis of morphological and physiological growth parameters of *A. spinosa* (AS) plants after 15.5 months of inoculation with *T. nivea* (TN)

## Additional Information and Declarations

### Field Study Permissions

The following information was supplied relating to field study approvals (*i.e.*, approving body and any reference numbers): The Tirmania nivea specimens collection was not subjected to any restriction or specific permissions. The ascomata were harvested from open lands that are not privately-owned or protected and no specific permissions were required for these collection sites by the local authorities.

### DNA Deposition

The following information was supplied regarding the deposition of DNA sequences: The Tirmania nivea rDNA ITS sequence is available in GenBank: MZ379289.

### Data Availability

The following information was supplied regarding data availability: The Fasta rDNA ITS sequence of Tirmania nivea, the raw data for daily germination rate and morphological and physiological growth parameters, and the statistical results are available in the Supplementary Files. The sequences are available in GenBank: MZ379289.

## References

[ref-1] Al-Laith AAA (2010). Antioxidant components and antioxidant/antiradical activities of desert truffle (*Tirmania nivea*) from various Middle Eastern origins. Journal of Food Composition and Analysis.

[ref-2] Al-Laith AAA, Kagan-Zur V, Roth-Bejerano N, Sitrit Y, Morte A (2014). Nutritional and antioxidant properties of the white desert truffle *Tirmania nivea* (Zubaidi). Desert Truffles: phylogeny, physiology, distribution and domestication.

[ref-3] Al-Rahmah AN (2001). Truffle of deserts and jungles.

[ref-4] Al-Ruqaie IM (2009). Effect of treatment process and preservation method on shelf life of truffles: II. Non-conventional methods (radiation). International Journal of Biological Chemistry.

[ref-5] Alessio GA, Peñuelas J, De Lillis M, Llusià J (2008). Implications of foliar terpene content and hydration on leaf flammability of *Quercus ilex* and *Pinus halepensis*. Plant Biology.

[ref-6] Alsheikh A, Molina R (1984). Mycorrhizae of annual *Helianthemum* species formed with desert truffles.

[ref-7] Alsheikh AM, Trappe JM (1983). Desert truffles: the genus *Tirmania*. Transactions of the British Mycological Society.

[ref-8] Altschul SF, Madden TL, Schäffer AA, Zhang J, Zhang Z, Miller W, Lipman DJ (1997). Gapped BLAST and PSI-BLAST: a new generation of protein database search programs. Nucleic Acids Research.

[ref-9] Augé RM (2001). Water relations, drought and vesicular arbuscular mycorrhizal symbiosis. Mycorrhiza.

[ref-10] Awameh MS (1981). The response of *Helianthemum salicifolium* and *H. ledifolium* to infection by the desert truffle *Terfezia boudieri*. Mushroom Science.

[ref-11] Awameh M, Alsheikh A, Al-Ghawas S (1979). Mycorrhizal synthesis between *Helianthemum ledifolium*, H. salicifolium and four species of the genera *Terfezia* and *Tirmania* using ascospores and mycelial cultures obtained from ascospore germination.

[ref-12] Bani-Aameur F, Alouani M (1999). Argan (*Argania spinosa* (L.) Skeels) seed viability and dormancy. Ecologia Mediterranea.

[ref-13] Barrs HD, Kozlowski TT (1968). Determination of water deficits in plant tissues. Water deficits and plant growth.

[ref-14] Benaouf Z, Miloudi A, Belkhodja M (2014). Germination tests of seeds of argan tree (*Argania spinosa* (l.) skeels) of two sources (Tindouf and Mostaganem) in the semi-arid western Algerian. African Journal of Plant Science.

[ref-15] Berka S, Harfouche A (2001). Influence of some physical and chemical treatments and of temperature on the faculty of germination of argan seeds. Revue Forestière Française.

[ref-16] Bokhary HA (1987). Desert truffles Al-kamah of the Kingdom of Saudi Arabia. I. Occurrence, identification and distribution. Arab Gulf Journal of Scientific Research.

[ref-17] Bokhary HA, Suleiman AAA, Basalah MO (1989). The fatty acid components of the desert truffle Al Kamah of Saudi Arabia. Journal of Food Protection.

[ref-18] Bouatia M, Touré HA, Cheikh A, Eljaoudi R, Rahali Y, Oulad Bouyahya Idrissi M, Khabar L, Draoui M (2018). Analysis of nutrient and antinutrient content of the truffle (*Tirmania pinoyi*) from Morocco. International Food Research Journal.

[ref-19] Chellal A, Lukasova E (1995). Evidence for antibiotics in two Algerien truffles *Terfezia* and *Tirmania*. Pharmazie.

[ref-20] Chevalier G, Kagan-Zur V, Roth-Bejerano N, Sitrit Y, Morte A (2014). The European Desert Truffles. Desert truffles: phylogeny, physiology, distribution and domestication.

[ref-21] Chevalier G, Dupré C, Riousset L, Dexheimer J (1984). Synthèse mycorhizienne entre *Terfezia leptoderma* Tul et diverses Cistacées. Agronomie.

[ref-22] Chevalier G, Grente J (1979). Application pratique de la symbiose ectomycorhizienne: production à grande échelle de plants mycorhizés par la truffe (*Tuber melanosporum* Vitt.). Mushroom Science.

[ref-23] Chevalier G, Grente J, Pollacsek A (1973). Obtention de mycorhizes de différents *Tuber* par synthèse à partir de spores en conditions gnotoxéniques et à partir de cultures pures de mycélium en conditions axéniques et gnotoxéniques. Annales de Phytopathologie.

[ref-24] Ching TM (1959). Activation of germination in Douglas fir seed by hydrogen peroxide. Plant Physiology.

[ref-25] Dafri A, Beddiar A (2018). Morphological characterisation of the mycorrhizal symbiosis between *Tuberaria guttata* (L.) Fourr and *Terfezia arenaria* (Moris) Trappe. Symbiosis.

[ref-26] Dexheimer J, Gerard J, Leduc JP, Chevalier G (1985). Etude ultrastructurale comparée des associations symbiotiques mycorhiziennes *Helianthemum salicifolium*-*Terfezia claveryi* et *Helianthemum salicifolium*-*Terfezia leptoderma*. Canadian Journal of Botany.

[ref-27] Elsayed EA, Alsahli FD, Barakat IA, El Enshasy HA, Wadaan MA (2019b). Assessment of *in vitro* antimicrobial and anti-breast cancer activities of extracts isolated from desert truffles in Saudi Arabia. Journal of Scientific Research.

[ref-28] Elsayed EA, Alsahli FD, Enshasy HAEl, Wadaan MA (2019a). Cytotoxic activities of different solvent extracts of *Tirmania nivea* and *Terfezia claveryi* against HepG2 and L929 Cells. Journal of Scientific Research.

[ref-29] Fortas Z (1990). Etude de trois espèces de terfez: caractères culturaux et cytologie du mycélium isolé et associé à *Helianthemum guttatum*. Thèse de Doctorat.

[ref-30] Fortas Z, Chevalier G (1992). Effet des conditions de culture sur la mycorhization de *l’Helianthemum guttatum* par trois espèces de terfez des genres *Terfezia* et *Tirmamia* d’Algérie. Canadian Journal of Botany.

[ref-31] Gargano ML, Bella P, Panno S, Arizza V, Inguglia L, Catara V, Venturella G, Davino S (2017). Antimicrobial activity of the extracts of *Terfezia claveryi* and *Tirmania pinoyi* against gram-positive and gram-negative bacteria causal agent of diseases in tomato. Chemical Engineering Transactions.

[ref-32] Gerdemann JW, Torrey JG, Clarkson DT (1975). Vesicular–arbuscular mycorrhizae. The development and function of roots.

[ref-33] Ghildiyal SK, Sharma CM, Khanduri VP (2007). Improvement in germination of chirpine (*Pinus roxburghii*) by a presowing treatment with hydrogen peroxide. Journal of Tropical Forest Science.

[ref-34] González L, González-Vilar M, Reigosa Roger MJ (2001). Determination of relative water content. Handbook of plant ecophysiology techniques.

[ref-35] Gouzi H, Belyagoubi L, Abdelali KN, Khelifi A (2011). *In vitro* antibacterial activities of aqueous extracts from Algerian desert truffles (*Terfezia* and *Tirmania*, Ascomycetes) against *Pseudomonas aeruginosa* and *Staphylococcus aureus*. International Journal of Medicinal Mushrooms.

[ref-36] Gouzi H, Leboukh M, Bouchouka E (2013). Antioxidant and antiradical properties of methanolic extracts from Algerian wild edible desert truffles (*Terfezia* and *Tirmania*, Ascomycetes). International Journal of Medicinal Mushrooms.

[ref-37] Gutiérrez A, Morte A, Honrubia M (2003). Morphological characterization of the mycorrhiza formed by *Helianthemum almeriense* Pau with *Terfezia claveryi* Chatin and *Picoa lefebvrei* (Pat.) Maire. Mycorrhiza.

[ref-38] Hamza A, Jdir H, Zouari N (2016). Nutritional, antioxidant and antibacterial properties of *Tirmania nivea*, a wild edible desert truffle from Tunisia arid zone. Medicinal and Aromatic Plants.

[ref-39] Hetrick BAD, Wilson GWT, Cox TS (1992). Mycorrhizal dependence of modern wheat varieties, landraces, and ancestors. Canadian Journal of Botany.

[ref-40] Honrubia M, Andrino A, Morte A, Kagan-Zur V, Roth-Bejerano N, Sitrit Y, Morte A (2014). Preparation and Maintenance of Both Man-Planted and Wild Plots. Desert Truffles: phylogeny, physiology, distribution and domestication.

[ref-41] Hussain G, Al-Ruqaie MA (1999). Occurrence, chemical composition, and nutritional value of truffles: an overview. Pakistan Journal of Biological Sciences.

[ref-42] Ikinci A (2014). Effects of the water presoaking duration and gibberellic acid treatments on seed germination of *Argania Spinosa* L. under nursery conditions. Fresenius Environmental Bulletin.

[ref-43] Imani A, Rasouli M, Tavakoli R, Zarifi E, Fatahi R, Barba-Espin G, Martinez-Gomez P (2011). Optimization of seed germination in *Prunus* species combining hydrogen peroxide or gibberellic acid pre-treatment with stratification. Seed Science and Technology.

[ref-44] Johnson NC, Graham JH, Smith FA (1997). Functioning of mycorrhizal associations along the mutualism–parasitism continuum. The New Phytologist.

[ref-45] Kagan-Zur V, Kuang J, Tabak S, Taylor FW, Roth-Bejerano N (1999). Potential verification of a host plant for the desert truffle *Terfezia pfeilii* by molecular methods. Mycological Research.

[ref-46] Khelifi L, Morsli A, Khelifi-Slaoui M (1996). Premiers résultats sur l’obtention *in vitro* de germinations d’Arganier *Argania spinosa* (L.) Skeel. Annales Agronomiques INA.

[ref-47] Kovács GM, Trappe JM, Kagan-Zur V, Roth-Bejerano N, Sitrit Y, Morte A (2014). Nomenclatural history and genealogies of desert truffles. Desert Truffles: phylogeny, physiology, distribution and domestication.

[ref-48] Kovács GM, Vágvölgyi C, Oberwinkler F (2003). *In Vitro* interaction of the truffle *Terfezia terfezioides* with *Robinia pseudoacacia* and *Helianthemum ovatum*. Folia Microbiologica.

[ref-49] Langeron K (1952). Précis de mycologie.

[ref-50] Leduc JP, Dexheimer J, Chevalier G, Gianinazzi-Pearson V, Gianinazzi S (1986). Etude ultrastructurale comparée des associations de *Terfezia leptoderma* avec *Helianthemum salicifolium*, Cistus albidus et *Cistus salviaefolius*. Aspects physiologiques et génétiques des mycorhizes. 1er Sem sur les mycorhizes.

[ref-51] Li ZG, Gong M, Liu P (2012). Hydrogen sulfide is a mediator in H_2_O_2_-induced seed germination in *Jatropha Curcas*. Acta Physiologiae Plantarum.

[ref-52] Mandeel QA, Al-Laith AAA (2007). Ethnomycological aspects of the desert truffle among native Bahraini and non-Bahraini peoples of the Kingdom of Bahrain. Journal of Ethnopharmacology.

[ref-53] Martins A, Siddiqui ZA, Akhtar MS, Futai K (2008). *In vitro* mycorrhization of micropropagated plants: studies on *Castanea sativa* Mill. Mycorrhizae: Sustainable agriculture and forestry.

[ref-54] Miloudi A, Belkhodja M (2009). The contribution to the research of the germination conditions of Argan seeds (*Argania spinosa* L. Skeels): the particular study of the water pre-soaking duration and the harvest year of seeds effects on the germination. European Journal of Scientific Research.

[ref-55] Morte A, Andrino A, Kagan-Zur V, Roth-Bejerano N, Sitrit Y, Morte A (2014). Domestication: preparation of mycorrhizal seedlings. Desert Truffles: phylogeny, physiology, distribution and domestication.

[ref-56] Morte A, Andrino A, Honrubia M, Navarro-Ródenas A, Zambonelli A, Bonito GM (2012). Terfezia cultivation in arid and semiarid soils. Edible ectomycorrhizal mushrooms.

[ref-57] Morte A, Gutiérrez A, Ródenas AN, Pérez-Moreno J, Guerin-Laguette A, Flores Arzú R, Yu FQ (2020). Advances in Desert Truffle Mycorrhization and Cultivation. Mushrooms, humans and nature in a changing world.

[ref-58] Morte A, Honrubia M, Gutiérrez A, Varma A (2008). Biotechnology and cultivation of desert truffles. Mycorrhiza: state of the art genetics and molecular biology, eco-function, biotechnology, eco-physiology, structure and systematics.

[ref-59] Morte A, Lovisolo C, Schubert A (2000). Effect of drought stress on growth and water relations of the mycorrhizal association *Helianthemum almeriense*-*Terfezia claveryi*. Mycorrhiza.

[ref-60] Morte A, Navarro-Ródenas A, Nicolas E (2010). Physiological parameters of desert truffle Mycorrhizal *Helianthemum almeriense* plants cultivated in orchards under water deficit conditions. Symbiosis.

[ref-61] Morte A, Pérez-Gilabert M, Gutiérrez A, Arenas F, Marqués-Gálvez JE, Bordallo JJ, Rodríguez A, Berná LM, Lozano-Carrillo C, Navarro-Ródenas A, Varma A, Prasad R, Tuteja N (2017). Basic and applied research for desert truffle cultivation. Mycorrhiza-eco-physiology, secondary metabolites, nanomaterials.

[ref-62] Morte A, Zamora M, Gutiérrez A, Honrubia M, Azcoń-Aguilar C, Barea JM, Gianinazzi S, Gianinazzi-Pearson V (2009). Desert truffle cultivation in Semiarid Mediterranean areas. Mycorrhizas—functional processes and ecological impact.

[ref-63] Navarro-Ródenas A, Bárzana G, Nicolás E, Carra A, Schubert A, Morte A (2013). Expression analysis of aquaporins from desert truffle mycorrhizal symbiosis reveals a fine-tuned regulation under drought. Molecular Plant-Microbe Interactions.

[ref-64] Navarro-Ródenas A, Pérez-Gilabert M, Torrente P, Morte A (2012). The role of phosphorus in the ectendomycorrhiza continuum of desert truffle mycorrhizal plants. Mycorrhiza.

[ref-65] Neggaz S, Fortas Z (2013). Tests of antibiotic properties of Algerian desert truffle against bacteria and fungi. Journal of Life Sciences.

[ref-66] Nouaim R, Mangin G, Breuil MC, Chaussod R (2002). The argan tree (*Argania spinosa*) in Morocco: propagation by seeds, cuttings and *in-vitro* techniques. Agroforestry Systems.

[ref-67] Owaid MN, Muslim RF, Hamad HA (2018). Mycosynthesis of silver nanoparticles using *Tirmania* sp. desert truffle, Pezizaceae, and their antibacterial activity. Jordan Journal of Biological Sciences.

[ref-68] Phillips J, Hayman D (1970). Improved procedures for clearing roots and staining parasitic and vesicular-arbuscular mycorrhizal fungi for rapid assessment of infection. Transactions of the British Mycological Society.

[ref-69] Plenchette C, Fortin JA, Furlan V (1983). Growth responses of several plant species to mycorrhizae in a soil of moderate P-fertility. Plant and Soil.

[ref-70] Roth-Bejerano N, Livne D, Kagan-Zur V (1990). Helianthemum-Terfezia relations in different growth media. New Phytologist.

[ref-71] Sawaya WN, Al-Shalhat A, Al-Sogair A, Mohammad M (1985). Chemical composition and nutritive value of truffles of Saudi Arabia. Journal of Food Science.

[ref-72] Schillaci D, Cusimano MG, Cascioferro SM, Di Stefano V, Arizza V, Chiaramonte M, Inguglia L, Bawadekji A, Davino S, Gargano M, Venturella G (2017). Antibacterial activity of desert truffles from Saudi Arabia against *Staphylococcus aureus* and *Pseudomonas aeruginosa*. International Journal of Medicinal Mushrooms.

[ref-73] Slama A, Fortas Z, Boudabous A, Neffati M (2010). Cultivation of an edible desert truffle (*Terfezia boudieri* Chatin). African Journal of Microbiology Research.

[ref-74] Stojkovic D, Reiss FS, Ferreira ICFR, Barros L, Glamoclija J, Ciric A, Nikolic M, Stevic T, Giveli A, Sokovic M (2013). Tirmania pinoyi: chemical composition, *in vitro* antioxidant and antibacterial activities and in situ control of *Staphylococcus aureus* in chicken soup. Food Research International.

[ref-75] Trouvelot A, Kough JL, Gianinazzi-Pearson V (1986). Mesure du taux de mycorhization VA d’un système radiculaire. Recherche de méthodes d’estimation ayant une signification fonctionnelle. Physiological and genetical aspects of mycorrhizae: proceedings of the 1st European symposium on mycorrhizae, Dijon, 1–5 July 1985.

[ref-76] Turgeman T, Ben-Asher Y, Roth-Bejerano N, Kagan-Zur V, Kapulnik Y, Sitrit Y (2011). Mycorrhizal association between the desert truffle *Terfezia boudieri* and *Helianthemum sessiliflorum* alters plant physiology and fitness to arid conditions. Mycorrhiza.

[ref-77] Turgeman T, Lubinsky O, Roth-Bejerano N, Kagan-Zur V, Kapulnik Y, Koltai H, Zaady E, Ben-Shabat S, Guy O, Lewinsohn E, Sitrit Y (2015). The role of pre-symbiotic auxin signaling in ectendomycorrhiza formation between the desert truffle *Terfezia boudieri* and *Helianthemum sessiliflorum*. Mycorrhiza.

[ref-78] Wellburn AR (1994). The spectral determination of chlorophylls *a* and *b*, as well as total carotenoids, using various solvents with spectrophotometers of different resolution. Journal of Plant Physiology.

[ref-79] White TJ, Bruns T, Lee S, Taylor J (1990). Amplification and direct sequencing of fungal ribosomal RNA genes for phylogenetics. PCR Protocols: A Guide to Methods and Applications.

[ref-80] Zaretsky M, Kagan-Zur V, Mills D, Roth-Bejerano N (2006). Analysis of mycorrhizal associations formed by *Cistus incanus* transformed root clones with *Terfezia boudieri* isolates. Plant Cell Reports.

[ref-81] Zhu XC, Song FB, Xu HW (2010). Arbuscular mycorrhizae improves low temperature stress in maize via alterations in host water status and photosynthesis. Plant and Soil.

[ref-82] Zitouni-Haouar FEH, Alvarado P, Sbissi I, Boudabous A, Fortas Z, Moreno G, Manjón JL, Gtari M (2015). Contrasted genetic diversity, relevance of climate and host plants, and comments on the taxonomic problems of the genus Picoa (Pyronemataceae, Pezizales). PLOS ONE.

[ref-83] Zitouni-Haouar FEH, Carlavilla JR, Moreno G, Manjon JL, Fortas Z (2018). Genetic diversity of the genus *Terfezia* (Pezizaceae, Pezizales): new species and new record from North Africa. Phytotaxa.

[ref-84] Zitouni-Haouar FEH, Fortas Z, Chevalier G (2014). Morphological characterization of mycorrhizae formed between three *Terfezia* species (desert truffles) and several *Cistaceae* and Aleppo pine. Mycorrhiza.

[ref-85] Zunzunegui M, Ain-Lhout F, Jáuregui J, Barradas MD, Boutaleb S, Álvarez Cansino L, Esquivias MP (2010). Fruit production under different environmental and management conditions of argan, Argania spinosa (L.). Journal of Arid Environments.

