# Supplementary material for: Growth promotion and mycorrhizal colonization of Argan (Argania spinosa (L.) Skeels) inoculated with the edible desert truffle Tirmania nivea (Desf.) Trappe
Source: PeerJ. 2022 Aug 17;10:e13769. doi: 10.7717/peerj.13769 (PMC9392452; doi:10.7717/peerj.13769)
Supplement: Supplemental Information 1 [file peerj-10-13769-s001.pdf]

***Tirmania nivea* rDNA ITS FASTA sequence MZ379289 obtained in the present work:**

TCAATGGGTCCTACCTGATCTGAGGTCACCCAGGATATAAATGAGTGAGTTAAGG
CAAGCACAATTATACTCAAACAAACGACTTTATTATTATTACGTCTGATTCAAGA
ACAAACCATACTGCCTATGAATTTCTGAAGGACAACTCTTTATTATAAGTCACCTC
TCCAAAGTTATCCAAGTATTTGCAAAAGAATACTTGAGTAGAAGGGAGCTGACGC
TCAGACAGGCATGCCCTACGGAATACCATAGGGCGCAATGTGCGTTCAAAGATTC
GATGATTCACGAGATTCTGCAATTCACATTACTTATCGCATTTCACTGCGTTCTTC
ATCGATGCAAGAGCCTAGAGATCCGTTGTTGAAAGTTTTATTTCATTGAAGCAAA
AGATTCAGACAATTTGTTTCAAATCAAGTTTTGATAGATTGGTCTTACCACTGGCA
ACTCTCTGGCCTGAGTTATTCACACAAGCCAAAGGTTCTCACACAAAAGGAAAAA
CATGTCCAGTGGAAGCAATAGGGTAGGTAAAACAATGGGATATAAAAAAAACTT
GTAGTTTTTCTTTAATGATCCTTCCGCAGGTTCACCTACGGAAACCTTGTTACGAC
TTTTACTTCCTCTAATTGGAACCAAGA
