# Supplementary material for: Growth promotion and mycorrhizal colonization of Argan (Argania spinosa (L.) Skeels) inoculated with the edible desert truffle Tirmania nivea (Desf.) Trappe
Source: PeerJ. 2022 Aug 17;10:e13769. doi: 10.7717/peerj.13769 (PMC9392452; doi:10.7717/peerj.13769)
Supplement: Supplemental Information 5 [file peerj-10-13769-s005.docx]

**Mycorrhizal association *Argania spinosa* /*Tirmania nivea***

Morphological growth parameters (height, leaf number, leaf length, shoot fresh weight "FW", shoot dry weight "DW" and root fresh weight "FW"), and physiological growth parameters (chlorophyll a content, chlorophyll b content, relative water content, leaf hydration,) of *A. spinosa* (AS) plants after 15 months and a half of inoculation with *T. nivea* (TN). The effects of treatment were compared using Student’s t-test, P <0.05, level of significance.

- **Plant height** (cm)

| ***t- value*** | ***Dl*** | ***P*** | ***Signification*** |
| --- | --- | --- | --- |
| **4.48367** | **18** | **0.000287157** | **HS** |

- **Leaf number**

| ***t- value*** | ***Dl*** | ***P*** | ***Signification*** |
| --- | --- | --- | --- |
| **3.86404** | **18** | **0.001136978** | **S** |

- **Leaf length** (cm)

| ***t- value*** | ***Dl*** | ***P*** | ***Signification*** |
| --- | --- | --- | --- |
| **2.77810** | **18** | **0.012404945** | **S** |

- **Shoot FW** (g)

| ***t- value*** | ***Dl*** | ***P*** | ***Signification*** |
| --- | --- | --- | --- |
| **2.82140** | **18** | **0.011305701** | **S** |

- **Shoot DW** (g)

| ***t- value*** | ***Dl*** | ***P*** | ***Signification*** |
| --- | --- | --- | --- |
| **3.34185** | **18** | **0.003629729** | **S** |

- **Root FW** (g)

| ***t- value*** | ***Dl*** | ***P*** | ***Signification*** |
| --- | --- | --- | --- |
| **3.31765** | **18** | **0.0038291234** | **S** |

- **Relative water content** (%)

| ***t- value*** | ***Dl*** | ***P*** | ***Signification*** |
| --- | --- | --- | --- |
| **3.39241** | **18** | **0.003245576** | **S** |

- **Leaf hydration** (%)

| ***t- value*** | ***Dl*** | ***P*** | ***Signification*** |
| --- | --- | --- | --- |
| **1.73154** | **18** | **0.100458863** | **NS** |

- **Chlorophyll a** (mg/g)

| ***t- value*** | ***Dl*** | ***P*** | ***Signification*** |
| --- | --- | --- | --- |
| **3.51427** | **18** | **0.002476999** | **S** |

- **Chlorophyll b** (mg/g)

| ***t- value*** | ***Dl*** | ***P*** | ***Signification*** |
| --- | --- | --- | --- |
| **4.23333** | **18** | **0.000499824** | **HS** |
